# Effects of Steam Sterilization on 3D Printed Biocompatible Resin Materials for Surgical Guides—An Accuracy Assessment Study

**DOI:** 10.3390/jcm9051506

**Published:** 2020-05-17

**Authors:** Neha Sharma, Shuaishuai Cao, Bilal Msallem, Christoph Kunz, Philipp Brantner, Philipp Honigmann, Florian M. Thieringer

**Affiliations:** 1 Department of Oral and Cranio-Maxillofacial Surgery, University Hospital Basel, Spitalstrasse 21, 4031 Basel, Switzerland; neha.sharma@usb.ch (N.S.); shuaishuai.cao@unibas.ch (S.C.); bilal.msallem@usb.ch (B.M.); christoph.kunz@usb.ch (C.K.); 2 Medical Additive Manufacturing Research Group, Department of Biomedical Engineering, University of Basel, Gewerbestrasse 16, 4123 Allschwil, Switzerland; philipp.brantner@usb.ch (P.B.); philipp.honigmann@ksbl.ch (P.H.); 3 Radiology Department, University Hospital Basel, Petersgraben 4, 4031 Basel, Switzerland; 4 Hand Surgery, Cantonal Hospital Basel-land, Rheinstrasse 26, 4410 Liestal, Switzerland

**Keywords:** 3D printing, additive manufacturing, biocompatible resin, dimensional accuracy, in-house, steam sterilization, surgical guide

## Abstract

Computer-assisted surgery with three-dimensional (3D) printed surgical guides provides more accurate results than free-hand surgery. Steam sterilization could be one of the factors that affect the dimensions of surgical guide resin materials, leading to inaccuracies during surgeries. The purpose of this study was to evaluate the effects of steam sterilization on the dimensional accuracy of indication-specific hollow cube test bodies, manufactured in-house using Class IIa biocompatible resin materials (proprietary and third-party). To evaluate the pre- and post-sterilization dimensional accuracy, root mean square (RMS) values were calculated. The results indicate that, in all the groups, steam sterilization resulted in an overall linear expansion of the photopolymeric resin material, with an increase in outer dimensions and a decrease in inner dimensions. The effects on the dimensional accuracy of test bodies were not statistically significant in all the groups, except PolyJet Glossy (*p* > 0.05). The overall pre- and post-sterilization RMS values were below 100 and 200 µm, respectively. The highest accuracies were seen in proprietary resin materials, i.e., PolyJet Glossy and SLA-LT, in pre- and post-sterilization measurements, respectively. The dimensional accuracy of third-party resin materials, i.e., SLA-Luxa and SLA-NextDent, were within a comparable range as proprietary materials and can serve as an economical alternative.

## 1. Introduction

In craniomaxillofacial surgery, the combined use of modern three-dimensional (3D) imaging and virtual planning software has provided tremendous help in the pre-operative planning of surgical interventions [1,2]. Such advents, when integrated with medical additive manufacturing (AM) or 3D printing, facilitates the transfer of virtual planning to the surgical site through patient-specific surgical/cutting guides [3,4,5,6]. Lately, several hospitals across the globe have integrated in-house 3D printing into their daily clinical practices to achieve personalized patient treatments [7,8].

In-house 3D printing of patient-specific surgical guides has been used in various complex craniomaxillofacial surgeries to increase precision, reduce morbidity, and operation time [9,10]. A 3D printed surgical guide is a medical device that provides a predefined osteotomy path during surgery and facilitates an accurate translation of the desired surgical treatment plan to reality [9]. As surgical guides are exposed to patient’s blood, bone, and oral tissues, these are categorized as “critical items” as per the guidelines for infection control published by the Centers for Disease Control and Prevention [11,12]. According to the guidelines published in “Additively Manufactured Medical Products—The FDA Perspective”, as with any external service provider, hospital-based or in-house 3D printing setups should provide the same safety and efficacy standards for medical devices to avoid infection [13]. With 3D printing gaining popularity, few hospitals have integrated quality assurance measures into their digital workflows to ensure optimal performance and accuracy of 3D printers and biomaterials [14,15].

Consistent with all other instruments, potentially entering a surgical field, biocompatibility and sterilization feasibility of 3D printed parts are some vital aspects that should be considered [16]. Invasive procedures that involve contact by a surgical guide with a patient’s sterile tissue or mucous membranes pose a high risk of infection if the surgical guide is contaminated with any microorganism. Therefore, sterilization of a 3D printed surgical guide is crucial to prevent any microbial contamination that could lead to infection in the patient [11]. This requirement to sterilize a 3D printed surgical guide further raises concerns about the dimensional accuracy of a surgical guide. If a surgical guide made with biocompatible resin material is deformed or modified during the sterilization procedure, it will affect the accuracy of a surgical procedure. Therefore, the sterilized surgical guide must be within a certain dimensional tolerance level. Generally, the sterilization of critical medical devices such as surgical guides can be achieved by autoclaving (steam under pressure), ionizing radiation (plasma and gamma radiations), dry heat, or heat/chemical vapor [17]. Sterilization methods with ionizing radiation or gas plasma are expensive and mainly used in tertiary care hospitals and medical centers. On the other hand, moist heat in the form of saturated steam under pressure (autoclaving or steam sterilization) is an inexpensive, high penetrating, fast-acting form of sterilization with easier accessibility in many hospitals [11]. This readily available means of sterilization acts as a convenient and practical solution to surgeons to sterilize the surgical guides for direct use in the operating room.

With the availability of in-house 3D printing, the fabrication of surgical guides has shifted from external service providers to clinicians’ hands. 3D printing of surgical guides in biocompatible materials is accomplished using photopolymeric resin materials [18,19]. With continuous advancements in 3D printing technology, clinicians can now select from an array of 3D printers and materials with certified biocompatibility properties [20,21]. 3D printers’ manufacturers are developing their printers and rendering their system open to other third-party materials available from other manufacturers. These third-party biocompatible resin materials are at a more economical price than proprietary (manufacturer’s standard) resin materials. This flexibility to choose among inexpensive biocompatible resin materials proposes an attractive option to clinicians and has gathered a considerable amount of attention at the point-of-care manufacturing. As off-site production and shipping of 3D printed surgical guides can be time-consuming and expensive, in-house fabrication of surgical guides can be completed within a brief turnaround time and at a much lower cost [18]. This transition opens recent developments and thus incites a need for standardized protocols that should be followed at the point-of-care manufacturing. Although biocompatible resin material manufacturers suggest sterilization feasibility of their material, there is little knowledge related to the effects of sterilization on the dimensional accuracy of parts manufactured with these materials.

Therefore, the purpose of the present study was to investigate the effects of steam sterilization on the dimensional accuracy of 3D printed test bodies fabricated in-house using proprietary (standard) and third-party biocompatible resin materials. This study involved two research questions:

1. Would steam sterilization result in a significant change in dimensions of biocompatible resin materials’ 3D printed test bodies?
2. Would the dimensional accuracy of test bodies be similar across all the groups, i.e., proprietary and third-party biocompatible resin materials?

## 2. Materials and Methods

### 2.1. Equipment and Materials

In this study, we chose six (three proprietary and three third-party) Class IIa medically certified biocompatible resin materials, commonly available in the market for the fabrication of surgical guides, splints, and drill templates. Depending on the resin material fabrication feasibility, two in-house available 3D printers were utilized. Group 1 and 2 test bodies were printed in a PolyJet desktop 3D printer (Objet 30 Prime^TM^, Stratasys Ltd., Minneapolis, MN, USA). Two proprietary photopolymer resins were used in the fabrication process, one as core material to build up test bodies (MED610, Stratasys Ltd., Minneapolis, MN, USA) and the other as a water-soluble support material (SUP705, Stratasys Ltd., Minneapolis, MN, USA). 

Unlike PolyJet desktop 3D printer, Stereolithography (SLA) desktop 3D printer (Form2, Formlabs Inc., Somerville, MA, USA) permits the use of third-party biocompatible resin materials under open mode (experimental mode). Group 3 was a proprietary (standard) Dental LT Clear resin material (Form2, Formlabs Inc., Somerville, MA, USA), while Group 4–6 were third-party resin materials, namely LuxaPrint Ortho Plus (DMG Chemisch-Pharmazeutische Fabrik GmbH, Hamburg, Germany), NextDent Ortho Clear (Nextdent B.V., Soesterberg, Netherlands), and Freeprint Ortho (Detax GmbH & Co. KG, Ettlingen, Germany), respectively. However, due to limited success and failures in the printing feasibility of Group 6 (third-party) biocompatible resin material with the in-house available SLA desktop 3D printer, this group was excluded from the study, resulting in five study groups. The various study groups and printing profile specifications used in this study are summarized in Table 1.

### 2.2. Computer-Aided Design (CAD) Modeling of Reference Test Body

**Figure 1:**
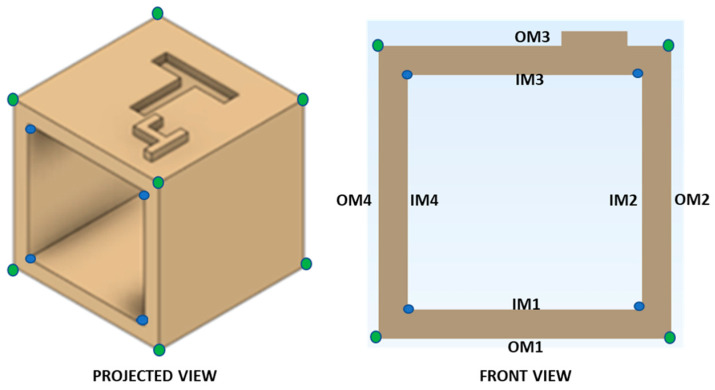
Projected and front view of a simplified experimental indication-specific test body design displaying the landmark points: blue points represent the landmark points for inner measurement (IM = 16 mm), while green points represent the landmark points for outer measurement (OM = 20 mm).

A calibration cube is the simplest benchmark object that provides multi-dimensional quantification of 3D printing inaccuracies in three axes. Therefore, as a reference for this study, an indication-specific test object was designed. The computer-aided design (CAD) of a simplified experimental test body is illustrated in Figure 1. Measuring organic shapes such as a surgical guide can be complicated, resulting in variable results; therefore, practical considerations were taken into account for the design of this experimental test body, such as a reproducible object for accurate measurements requiring a minimal amount of material and printing time. Test bodies with outer wall linear dimensions of 20 mm, inner wall linear dimensions of 16 mm, and a wall thickness of 2 mm were designed (Autodesk Fusion 360, v. 2.0.3803). For full quantification of analysis, this design helped in precise identification of measurement points along with the acquisition of several linear dimensional measurements within each test body. The linear dimensional measurements selected were 20 landmark points at the corner points (Figure 1). These measurements were classified into three groups: outer measurement (OM), inner measurement (IM), and overall measurement. The OM was intended to measure an expansion or contraction of the outer edges of the test body, and the IM was intended to reflect the similar changes in the inner angular planes. The overall measurement was intended to measure the distances of OM and IM features, and the overall shape of the test body. It was also used to analyze the overall phenomenon in which a test body deforms outward or inwards with steam sterilization. As the build platform of SLA desktop 3D printer is smaller in dimensions (X × Y × Z: 145 mm × 145 mm × 175 mm) compared to PolyJet desktop 3D printer (X × Y × Z: 294 mm × 192 mm × 148.6 mm), the number of test bodies printed was defined based on the size of SLA printer’s build platform. A vertical line and a horizontal line, intersecting at the middle part of the SLA square build platform, were virtually defined. The corner points, along with the intersecting points on the virtual square build platform, were selected, which represented the number of total test bodies fabricated. Hence, nine tests bodies per group were designed, each assigned with a number (T1–T9, in ascending order), on one of the test body face (intaglio surface). For 3D printing, test bodies’ CAD files were then exported in a standard tessellation language (STL) file format.

### 2.3. 3D Printing of Test Bodies

The STL files of test bodies were imported into the slicing software of the respective 3D printer. In the PolyJet desktop 3D printer, given that only manufacturer’s proprietary resin materials were used, the fabrication sequence was arbitrarily selected with the printing of Group 1 test bodies first, followed by Group 2 test bodies. The automatic placement feature in 3D printer’s slicer software (Objet Studio Print Wizard Software v. 9.2.11.6825) was used, suggesting the best orientation of test bodies. 

In the SLA desktop 3D printer, a standardized sequence of test bodies printing was employed with Group 3, i.e., proprietary resin material being printed first, followed by printing of the third-party resin materials Group 4 and 5, respectively. This was employed to reduce the chances of printout inaccuracy due to any potential “technical” variability. The respective slicing software (PreForm Software v. 2.14.1) was used, and the test bodies were oriented at an angle of 45° with the intaglio surface facing away from the build platform. This orientation for test bodies was selected to minimize the generation of peel forces during their detachment. Supports were generated using the auto-generation feature in this software.

### 2.4. Post-Processing and Post-Curing Processes of 3D Printed Test Bodies

**Figure 2:**
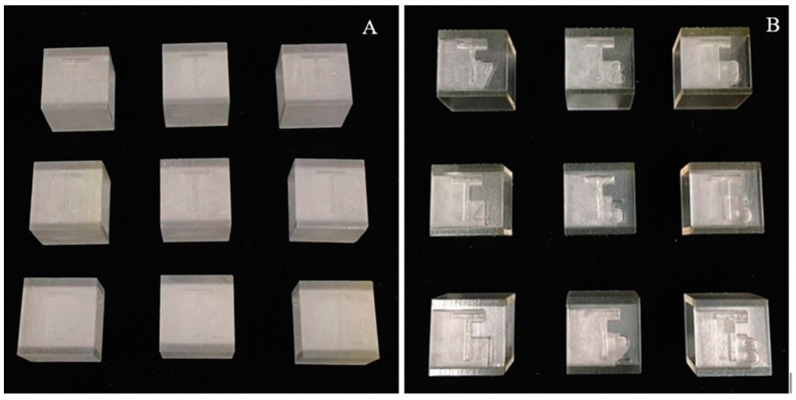
3D printed test bodies after removal of support material: (**A**) Group 1 (PolyJet Matte) test bodies (T1–T9); and (**B**) Group 2 (PolyJet Glossy) test bodies (T1–T9).

After the 3D printing processes were completed, test bodies were visually inspected for any defects or errors by an independent observer. For post-processing in Group 1 and 2 PolyJet printed test bodies, clay-sculpting tools were initially used to remove the support material, followed by cleaning in a WaterJet station. In WaterJet station, two spraying mechanisms were employed: wide-set spray, in which the spray nozzle lifts off large quantities of support material from the flat surfaces by cutting through the support material, and concentrated point spray, in which the nozzle clears out support from cavities by creating a “pilot hole” through the support material. No further post-curing processes were required in these groups′ test bodies (Figure 2).

**Figure 3:**
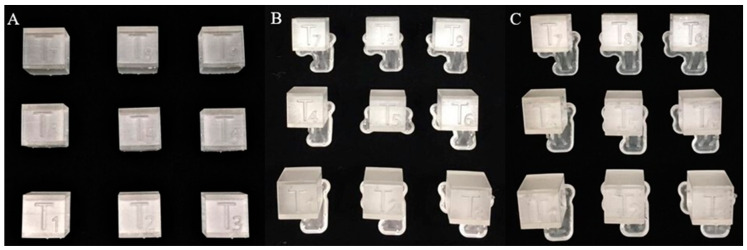
3D printed test bodies in a cured state: (**A**) Group 3 (SLA-LT) test bodies (T1–T9) after removal of support structures; (**B**) Group 4 (SLA-Luxa) test bodies (T1–T9) before removal of support structures; and (**C**) Group 5 (SLA-NextDent) test bodies (T1–T9) before removal of support structures.

Test bodies fabricated in the SLA desktop 3D printer (Figure 3) were first washed via Form Wash (Formlabs, Inc., Somerville, MA, USA)—circulating isopropyl alcohol (IPA) bath used to precisely stir and remove any uncured resin from print surfaces. A concentration of 90% IPA was used for the post-print washing procedure. To attain proper mechanical properties of each biocompatible resin material, additional post-curing procedures were done via a heated, rotating UV curing chamber (UVCA 2000; EnvisionTEC GmbH, Gladbeck, Germany) according to manufacturer’s instructions. This was followed by manual removal of support structures using fine cutting pliers.

### 2.5. Sterilization Method

All test bodies underwent a steam sterilization (autoclave) protocol at a temperature of 121 °C (250 °F) at 100 kPa (15 psi) for 15 minutes, as recommended by 3D printer’s resin manufacturers.

### 2.6. Data Collection and Assessment of Dimensional Accuracy

The dimensional accuracy of test bodies represents an important aspect and encompasses precision and trueness, which were analyzed according to ISO 5725-1 guidelines [22]. Precision or reproducibility refers to the closeness of two or more measurements to each other. It represents the extent of difference of different measurements from each other, which means the higher the precision is, the more similar different measurements are. On the other hand, trueness refers to the closeness between the measured value and the known standard dimension (CAD reference value) of the object. High trueness represents a result that is close or equal to the actual dimensions of the measured object [23].

The pre-sterilization measurements of test bodies were conducted with an electronic precision digital caliper (Vogel Germany GmbH & Co., KG, Kevelaer, Germany) with an accuracy of ±20 µm and a resolution of 100 µm. Each OM was the linear distance between two endpoints on the outer edge of the test body, whereas IM was on the inner surface. Thus, 12 different OM and eight different IM were taken, resulting in 20 linear overall measurements per test body or, for all test bodies (*n* = 9; T1–T9), 180 linear measurements for each group. Along with these measurements, dimensional differences in linear OM and IM measurements, between 3D printed test body and CAD reference model, were computed. For each value of the dimensional difference in OM, IM, and overall measurements, root mean square (RMS) values for dimensional accuracy (precision and trueness) were calculated by using the following formula:

(1) $$RMS=\frac{1}{\sqrt{n}}\cdot \sqrt{\overset{n}{\underset{i=1}{\sum }}{\left(r_{i}-o_{i}\right)}^{2}},$$

where $r_{i}$ is the measurement value of *i* linear dimension in the CAD reference, $o_{i}$ is the observed measurement value of *i* in the test body, and *n* is the total number of measurements. The RMS value is defined as the overall error, which serves as a measurement indicator of how far the deviations vary from zero between two datasets. Lower RMS values represent higher dimensional accuracy and vice versa. The post-sterilization data collection followed the same protocol as the pre-sterilization data collection.

### 2.7. Statistical Analysis

Descriptive statistics were used for the test bodies in all the groups. To evaluate the quantitative data distribution regarding the dimensional accuracy of the test bodies, the RMS values were calculated for each group. The data were represented as mean, standard deviation, median, and first and third quartile ranges. The Shapiro–Wilk test was applied to verify the data distribution of the RMS values of the test groups. A paired t-test was applied to compare the mean deviation in pre- and post-sterilization measurements within the same group. The intragroup and intergroup differences were analyzed using one-way analysis of variance (ANOVA), and Tukey’s HSD post hoc tests were performed after a statistically significant result to confirm where the difference occurred between groups. Data were collected and tabulated in Microsoft Excel 2016, and statistical analyses were performed using GraphPad Prism 7.0 (GraphPad Software, La Jolla, CA, USA). The significance criterion was set at α = 0.05.

## 3. Results

**Figure 4:**
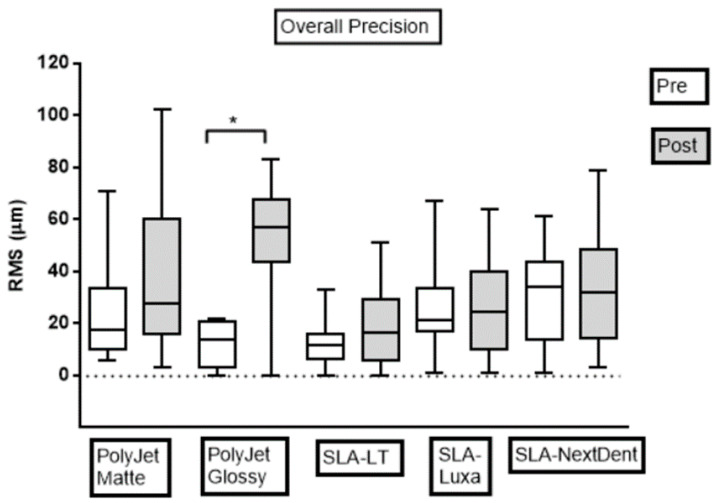
Boxplot demonstrating dimensional accuracy measurements (pre- and post-sterilization) reflecting overall precision RMS values in all groups. Pre, pre-sterilization; Post, post-sterilization; RMS, root mean square. The dotted line represents the CAD reference line.

**Figure 5:**
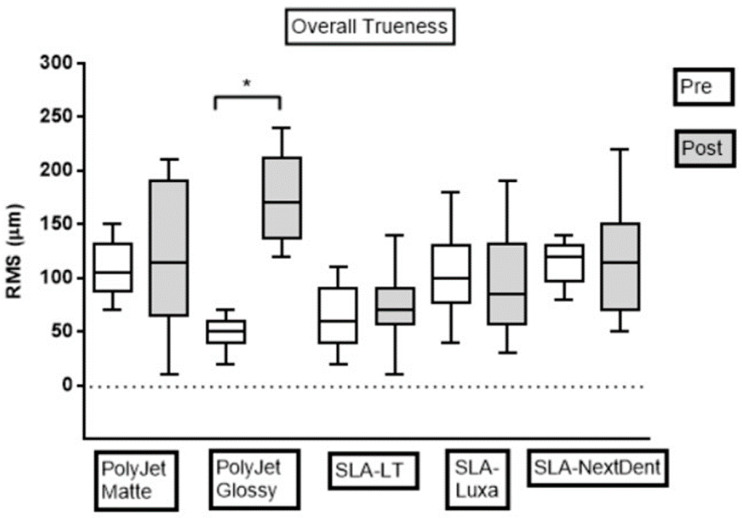
Boxplot demonstrating dimensional accuracy measurements (pre- and post-sterilization) reflecting overall trueness RMS values in all groups. Pre, pre-sterilization; Post, post-sterilization; RMS, root mean square. The dotted line represents the CAD reference line.

For the quantitative assessment of the dimensional accuracy of the test bodies within each group, RMS values were calculated. The evaluation of overall pre- and post-sterilization dimensional accuracy for precision and trueness in each group are shown in Figure 4 and Figure 5, respectively. The results indicate that, in all the groups, except Group 2 (PolyJet Glossy), steam sterilization displayed no statistically significant difference in dimensional accuracy of test bodies (*p* > 0.05). Statistically significant differences were seen in the dimensional accuracy of Group 2 (PolyJet Glossy) test bodies with steam sterilization concerning precision and trueness (*p* < 0.05). Test bodies in all the groups revealed higher dimensional accuracy in the pre-sterilization measurements as compared to post-sterilization measurements. Additionally, a similar pattern with a tendency to increase in OM and decrease in IM was noticed across all the groups with steam sterilization. The RMS values in pre- and post-sterilization accuracy assessment in all the groups were below 100 and 200 µm, respectively.

Furthermore, the intergroup comparison revealed statistically significant differences among the groups (*p* < 0.05). The comprehensive analyzes regarding pre- and post-sterilization dimensional accuracy, i.e., precision and trueness RMS values for all the groups, are described in the following sections.

### 3.1. Precision Assessment

For overall measurements in the pre-sterilization phase, RMS values (mean ± SD) for precision in PolyJet Glossy (12.3 ± 7.9 µm) and SLA-LT (13.3 ± 9.6 µm) were statistically different from SLA-NextDent (29.5 ± 17.9 µm) (*p* < 0.05) (Table 2 and Table 3). However, no statistically significant difference was observed in RMS values for precision in OM for SLA-LT (8.5 ± 4.1 µm) and PolyJet Glossy (9.25 ± 8.4 µm) and in IM among PolyJet Matte (14.5 ± 11.3 µm), PolyJet Glossy (16.88 ± 4.4 µm), and SLA-LT (20.5 ± 11.2 µm). Concerning precision in OM, SLA-LT (8.5 ± 4.1 µm) had the lowest and PolyJet Matte (31.0 ± 20.1 µm) had the highest RMS values. For precision in IM, PolyJet Matte (14.5 ± 11.3 µm) and SLA-NextDent (38.4 ± 15.2 µm) had the lowest and the highest RMS values, respectively. The overall precision for all groups in pre-sterilization measurements was ranked in the following descending order: PolyJet Glossy, SLA-LT, PolyJet Matte, SLA-Luxa, and SLA-NextDent.

For overall measurements in the post-sterilization phase, RMS values (mean ± SD) for precision in SLA-LT (18.3 ± 14.2 µm) and SLA-Luxa (25.3 ± 17.8 µm) differed significantly from PolyJet Glossy (51.0 ± 25.4 µm) (*p* < 0.05) (Table 3 and Table 4). There was no statistically significant difference in RMS values for precision in OM for PolyJet Matte (50.8 ± 29.7 µm) and SLA-NextDent (46.0 ± 19.4 µm). In IM, PolyJet Glossy (70.5 ± 8.0 µm) revealed a statistically significant difference as compared to other groups. Regarding precision in OM, SLA-LT (16.8 ± 12.1 µm) had the lowest and PolyJet Matte (50.8 ± 29.7 µm) had the highest RMS values. For precision in IM, SLA-Luxa (10.3 ± 6.4 µm) and PolyJet Glossy (70.5 ± 8.0 µm) had the lowest and the highest RMS values, respectively. The overall precision for all groups in post-sterilization measurements was ranked in the following descending order: SLA-LT, SLA-Luxa, SLA-NextDent, PolyJet Matte, and PolyJet Glossy. A composite matrix statistical analysis for overall pre- and post-sterilization precision among different groups is shown in Table 3.

### 3.2. Trueness Assessment

For overall measurements in the pre-sterilization phase, RMS values (mean ± SD) for trueness in PolyJet Glossy (47.8 ± 13.9 µm) and SLA-LT (61.7 ± 27.5 µm) were statistically different from SLA-Luxa (102.8 ± 37.7 µm), PolyJet Matte (108.9 ± 26.5 µm), and SLA-NextDent (115.0 ± 20.6 µm) (*p* < 0.05) (Table 2 and Table 5). There was no statistically significant difference in RMS values for trueness in OM, for PolyJet Glossy (41.1 ± 16.2 µm) and SLA-LT (48.9 ± 12.7 µm). In IM, RMS value for trueness in SLA-NextDent (128.9 ± 10.5 µm) differed significantly from the rest of the groups. Concerning trueness in OM, PolyJet Glossy (41.1 ± 16.2 µm) had the lowest and PolyJet Matte (132.2 ± 13.9 µm) had the highest RMS values. For trueness in IM, PolyJet Glossy (54.4 ± 7.3 µm) and SLA-NextDent (128.9 ± 10.5 µm) had the lowest and highest RMS values, respectively. The overall trueness for all groups in pre-sterilization measurements was ranked in the following descending order: PolyJet Glossy, SLA-LT, SLA-Luxa, PolyJet Matte, and SLA-NextDent.

For overall measurements in the post-sterilization phase, RMS values for trueness in PolyJet Glossy (176.7 ± 41.0 µm) differed significantly from the rest of the groups (*p* < 0.05) (Table 4). Fortrueness in OM and IM, SLA-LT (72.2 ± 19.9 µm) and PolyJet Glossy (214.4 ± 15.1 µm) differed statistically from the rest of the groups, respectively. However, no statistically significant difference was observed in RMS value for trueness in OM for SLA-Luxa (127.8 ± 33.1 µm) and PolyJet Glossy (138.9 ± 11.7 µm) groups. For trueness in OM, SLA-LT (72.2 ± 19.9 µm) had the lowest and PolyJet Matte (186.7 ± 18.0 µm) had the highest RMS values. For trueness in IM, SLA-Luxa (61.1 ± 22.1 µm) and PolyJet Glossy (214.4 ± 15.1 µm) had the lowest and highest RMS values, respectively. The overall trueness for all groups in post-sterilization measurements was ranked in the following descending order: SLA-LT, SLA-Luxa, SLA-NextDent, PolyJet Matte, and PolyJet Glossy. A composite matrix representation of statistical analysis for overall pre- and post-sterilization trueness among different groups is displayed in Table 5.

### 3.3. Deviations Distribution Pattern of Test Bodies regarding Position on 3D Printer’s Build Platform

**Figure 6:**
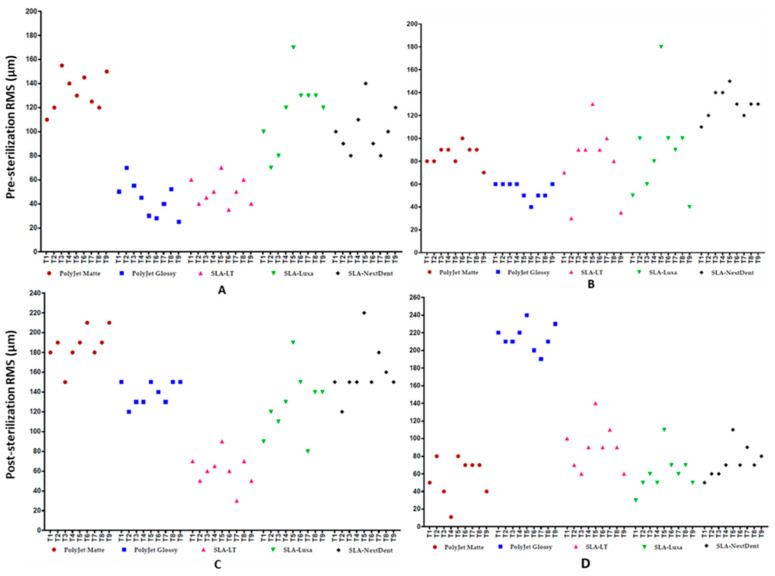
Scatter plot demonstrating the deviations distribution pattern of test bodies regarding their position on 3D printer’s build platform: (**A**) pre-sterilization outer measurements; (**B**) pre-sterilization inner measurements; (**C**) post-sterilization outer measurements; and (**D**) post-sterilization inner measurements.

The intragroup results reveal no statistically significant difference in the RMS values of test bodies within the same group in both PolyJet and SLA 3D printed test bodies (*p* > 0.05). However, in SLA 3D printer, the T5 test body (printed at the center/middle of the build platform) revealed an increased deviation distribution pattern. A scatter plot depicting the distribution of the deviations for each test body (T1–T9) with respect to its position on the respective 3D printer’s build platform is shown in Figure 6.

## 4. Discussion

The current study investigated the influence of steam sterilization on the dimensional accuracy of test bodies fabricated in Class IIa biocompatible (proprietary and third-party) resin materials. The study showed no significant influence of steam sterilization on the change in dimensions of test bodies within all groups except Group 2 (PolyJet Glossy). The mean deviations (RMS values) in dimensional accuracy in all the groups with steam sterilization were under 200 µm. Several studies have compared and assessed the dimensional accuracy of different 3D printing technologies [24,25,26,27,28,29,30,31]. These studies have defined a parameter of an error of 0.20–0.50 mm as a clinically acceptable range of differences [32,33]. In the present study, steam sterilization resulted in an overall linear expansion of the resin material in all the groups. Additionally, the current results reveal that, even though statistically significant differences were found among the study groups, the dimensional accuracy of test bodies fabricated using third-party biocompatible resin materials were comparable and within the same range as the proprietary biocompatible resin materials.

A comparison between the data in our study depicted an overall trend for higher dimensional accuracy in pre-sterilization measurements in all the groups (Table S1, Supplementary Data). The highest and lowest dimensional accuracies were observed in Group 2 (PolyJet Glossy) in the pre- and post-sterilization measurements, respectively. On the other hand, Group 3 (SLA-LT) displayed the highest dimensional accuracy in the post-sterilization measurements. In all except Group 4 (SLA-Luxa), a greater offset error change in dimensional accuracy was seen post-sterilization. Besides, a comparable variation with a tendency to increase in OM and a decrease in IM was observed in all the test bodies. The test bodies in all the groups had an overall expansion with respect to their shape. This expansion of biocompatible resin material on the inner side is more detrimental in surgeries than an expansion on the outer side. With in-house 3D printing gaining momentum, clinicians should, therefore, also focus on the errors made during the virtual surgical planning phase of a surgical guide. The key point is that the inner surfaces of the surgical guide should fit the individual patient’s bone surfaces perfectly and allow the insertion of metal sleeves or inserts [34]. Therefore, to compensate for the contraction in the guide’s internal surface with sterilization, along with printer’s calibration, an offset to the fitting part should be considered in the design phase of a surgical guide.

In hospitals with point-of-care manufacturing, the most widely used sterilization method is steam sterilization, in which sterilization occurs in an enclosed space (autoclave), at high pressure with the help of saturated steam [35]. Several articles can be found related to the accuracy evaluation in computer-guided surgeries [36,37], yet very few of them fully describe the effects of sterilization. The accuracy of computer-guided surgeries relies on all the cumulative and interactive errors, from medical imaging data acquisition, segmentation, virtual planning, 3D printing, and post-processing procedures to the surgical procedure [38]. Therefore, the clinical applicability of surgical guide materials with post-processing procedures such as sterilization is an essential aspect of evaluation. Oth et al. [39] investigated the morphological deformations induced by hydrogen peroxide on 3D printed genioplasty guides made from Polylactic Acid (PLA) and Polyethylene Terephthalate Glycol (PETG). It was concluded that steam sterilization was associated with higher morphological deformity than hydrogen peroxide sterilization. Such deformations were explained as PLA and PETG being low-temperature thermoplastics that cannot withstand steam sterilization, leading to polymer degradation and, therefore, hydrogen peroxide sterilization method is more appropriate. However, this finding was inconsistent with the study of Boursier et al. [40], who concluded that PLA printed objects could be sterilized in an autoclave. Therefore, for invasive surgeries with 3D printed medical devices, in addition to biocompatibility properties of a biomaterial, non-corrosiveness and heat-resistant properties should also be considered in regards to the method of sterilization.

Shaheen et al. [41] investigated the effect of steam sterilization and hydrogen peroxide gas plasma sterilization on PolyJet 3D printed objects. They concluded that higher morphological deformity was observed with steam sterilization, making it less reliable than gas plasma. Our results are different from the study by Shaheen et al. Despite the high temperature of 121 °C in an autoclave, none of the sterilized test bodies displayed major deformations, discolorations, or structural changes in our study. Moreover, our results are comparable with the study by Marie et al. [11]. They conducted a pilot study to investigate the effect of steam heat sterilization on the dimensional changes of surgical guides fabricated in Class I biocompatible resin material. It was concluded that there was no significant influence of steam heat sterilization on the dimensional changes of the tested parts. Our results also find that there was no clinically relevant dimensional change of test bodies after steam sterilization. Furthermore, our study investigated five Class IIa biocompatible resin materials from different manufacturers, and none of the test bodies displayed deformations.

PolyJet and SLA 3D printed photopolymeric parts have high mechanical isotropy due to densely compact resin volume. However, residual stresses might develop due to the thermal expansion and contraction of the resin material during the polymerization stage, which might lead to the deformation of the printed part. For SLA printing, the stiffness and strength of parts can be affected by varying the wavelengths and temperatures of the laser beam during curing, while, in PolyJet printing, the ultraviolet (UV) exposure time has a significant effect on mechanical properties [42]. Similar to advanced dental restorative materials [43], the mechanical properties of photopolymeric 3D printed materials are also dependent upon the surface roughness, with smoother finished parts displaying improved mechanical strength properties [42].

Dimensional accuracy in 3D printing is multi-factorial and depends on several factors such as printing technology, post-processing methods, usage of support material, and object orientation [14,44]. The accuracy of PolyJet 3D printing is considered higher than SLA 3D printing technology [45]. However, to use a 3D printed part, the level of accuracy required is dependent on the clinical application, and it should be evaluated to the defined clinical tolerances level. According to our results, all groups of test bodies were within the defined relevant range. Group 2 (PolyJet Glossy) fabricated in PolyJet desktop 3D printer had the highest accuracy in pre-sterilization measurements, while, in the post-sterilization measurements, Group 3 (SLA-LT) fabricated in SLA desktop 3D printer had the highest accuracy. One explanation for this observation could be the difference in biocompatible resin material properties, which are affected by steam sterilization, and, therefore, play a role in dimensional accuracy. Furthermore, a significantly greater offset error change was observed with sterilization in Group 2 (PolyJet Glossy) test bodies. This difference can be explained due to the shape of the test body and the usage of support material required during the fabrication process of these test bodies. In Group 2 test bodies, dissolvable support material was only added where needed, such as inside the hollow cavity. As these test bodies were exposed to air during curing, where there was no support material, these parts were also susceptible to getting rounded edges during printing. These changes were markedly depicted post-sterilization, where slight rounding of test bodies at external and internal 90° corners were observed, resulting in higher RMS values. Nonetheless, the authors believe that a targeted evaluation of this specific group printing profile may provide a more robust analysis, and further quantification of this effect should be addressed in future studies. Therefore, according to our findings for PolyJet printed test bodies, usage of Group 1 (PolyJet Matte) is recommended when the test object has sharp, unified corners and edges, and requires a uniform finish. At the same time, Group 2 (PolyJet Glossy) is beneficial in situations where support removal might ruin the object’s tiny features, for the reduction in material costs and print time, and in printing intricate and complex anatomical geometries where more transparent features are required. From the support material removal perspective, Group 2 test bodies provided a much faster support removal than Group 1 counterparts.

Unlike PolyJet technology, support structures are not dissolvable in SLA 3D printing technology. Therefore, other parameters, such as the object’s position, orientation, and support structures, play an essential role in dimensional accuracy. Unkovskiy et al. [46] assessed the dimensional accuracies of SLA 3D printed objects to the object’s build orientation and position and concluded that objects oriented at a 45° angle and printed in the middle of the build platform had the highest dimensional accuracy. In our study, SLA 3D printed test bodies were printed at 45° orientation angle at nine different positions. Although no statistically significant differences were observed in our study, the T5 test body, i.e., printed at the center/middle of the build platform, displayed maximum deviations. One explanation for this observation is the clouding of the polydimethylsiloxane (PDMS) layer in the SLA printer’s resin tank at this position. The authors would like to emphasize that the repeated habitual placement of the object at the same position in an SLA 3D printer may potentially result in clouding/fogging of the PDMS (silicone) layer in the resin tank. This can eventually result in an under-cured and failed print at that position. Although we did not experience any failed prints, the high deviations noticed in the T5 test body signify that the test body was not fully polymerized due to weak laser beam intensity in that region. Therefore, to extend the life of the SLA 3D printer, it is essential that the object’s placement is varied and distributed over the entire platform. The orientation of the test body guides the placement and configuration of non-dissolvable support structures. Therefore test bodies in the SLA 3D printer were oriented at an angle to avoid the generation of support structures where the measurements were taken. Orienting a planar surface flat to the build platform results in a greater cross-sectional area attaching to the PDMS (silicone) layer of the resin tank and require higher peel forces for detachment. These forces can distort the inter-layer bonding between the thin resin layers and detach the object from the build platform during printing [14]. Moreover, orienting hollow cavities such as open slots of a surgical/cutting guide, facing towards the resin tank, will result in resin and air entrapment within the object. These entrapments act as suction forces inside the guide and can cause print failure. Furthermore, support structures placed inside the guiding/fitting surface of a surgical guide can leave remnants after removal. Hence, the strategic orientation of surgical guide for support structures should be considered avoiding slight cumulation of errors, potentially resulting in surgical inaccuracies.

Maintaining efficacy and safety in an in-house 3D printing is of paramount importance. Clinicians who create 3D printed parts should understand the various factors and pitfalls that can affect an object’s reproducibility and accuracy [13]. Therefore, operational and regulatory measures should be undertaken to assess whether the intended 3D printed part conforms to its clinical use. Besides, quality control programs should be a part of regular protocols for in-house 3D printing labs undergoing regular accuracy testing, recalibration, and maintenance procedures [14]. One such component of a quality program is the verification of part accuracy [47], which was explored in this study. There are various strategies for part verification, such as manual measurements and measurements via surface imaging strategies (3D surface scanning). We resorted to manual measurements, seeing the best-fit option for the indication-specific test body used in our study. We learned that the test body material color profoundly influenced the utilization of measurement method. As the test bodies were fabricated in the clear (transparent) resin material, we were unable to obtain complete datasets through a surface scanner. Spraying the test bodies with white reflective paint or powder might have helped the scanning procedure. Still, we were concerned that this method would have affected the overall geometry, and, due to this reason, surface scanning was not implemented in this study. On the other hand, manual measurements provided a straightforward and cost-effective strategy. Therefore, to assure the quality of 3D printing in-house at all times, such a test object study can be executed easily and should be exercised periodically.

We state, however, in the present study, an isosymmetric shaped test body was used, and further evaluation of dimensional changes for complex surgical guides based on anatomical structures is imperative for future studies. Besides, we did not analyze the effects of steam sterilization concerning the mechanical properties of these biocompatible resin materials. Further studies are required to assess these changes. Nevertheless, the authors believe that this study presents a comprehensive analysis of the change in dimensional accuracy with steam sterilization using various biocompatible resin materials in an in-house 3D printing facility.

Currently, in craniomaxillofacial surgery, the application of photocuring 3D printing technologies is limited to short-term and transient contact with the living body. However, technological advancements in photocuring based 3D printing technologies have enabled living cells to be included in the printing process itself, resulting in spatial positioning of living cells together with desired biomaterials and supporting biochemical factors within a 3D structure [48,49]. With the fabrication of nanocomposite-based 3D volumetric and functionalized scaffolds [50], these technologies have remarkable potential in the field of tissue engineering and regenerative medicine.

## 5. Conclusions

When it comes to the reliability of outputs from any technique that is eventually going to be used for a patient, the most critical parameter is its consistency in accuracy. Within the limitation of our study, the results underline the fact that steam sterilization results in an overall linear expansion of the photopolymeric resin materials used for surgical guides. This resulted in an increase in the outer dimensions and a decrease in the inner dimensions of the test bodies. The effects on the dimensional accuracy of test bodies were not statistically significant in all the groups except Group 2 (PolyJet Glossy). None of the 3D printed test bodies displayed deformations or discolorations with steam sterilization. The highest dimensional accuracy was observed in proprietary resin materials, i.e., Group 2 (PolyJet Glossy) and Group 3 (SLA-LT), in pre- and post-sterilization measurements, respectively. Additionally, the findings reveal that, even though statistically significant differences were found among the study groups, the dimensional accuracy of test bodies fabricated using third-party biocompatible resin materials were within a comparable range as proprietary biocompatible resin materials and these materials can serve as an economical alternative to clinicians with in-house 3D printing setup. 

## Figures and Tables

**Table 1:** Study groups and printing profile specifications.

Group	Resin Code Name	Printing Profile Setting
1	PolyJet Matte	Matte profile
2	PolyJet Glossy	Glossy profile
3	SLA-LT	Dental LT resin profile
4	SLA-Luxa	Clear V2 resin-experimental profile
5	SLA-NextDent	Dental SG resin-experimental profile

**Table 2:** Pre- sterilization RMS values (mean ± SD) (µm) of precision and trueness for outer, inner, and overall measurements of test bodies fabricated using different biocompatible resin materials.

		PolyJet Matte	PolyJet Glossy	SLA-LT	SLA-Luxa	SLA-NextDent
	Measurement	Mean ± SD	Mean ± SD	Mean ± SD	Mean ± SD	Mean ± SD
Precision	OM	31.0 ± 20.1 ^a^	9.25 ± 8.4 ^b^	8.5 ± 4.1 ^b^	26.7 ± 21.3 ^a,b^	23.5 ± 17.7 ^a,b^
	IM	14.5 ± 11.3 ^a^	16.88 ± 4.4 ^a^	20.5 ± 11.2 ^a^	23.4 ± 10.8 ^a,b^	38.4 ± 15.2 ^b^
	Overall	24.4 ± 18.7 ^a,b^	12.3 ± 7.9 ^a^	13.3 ± 9.6 ^a^	25.4 ± 17.5 ^a,c^	29.5 ± 17.9 ^b,c^
Trueness	OM	132.2 ± 13.9 ^a^	41.1 ± 16.2 ^b^	48.9 ± 12.7 ^b^	116.7 ± 30.0 ^a,c^	101.1 ± 19.0 ^c^
	IM	85.6 ± 8.8 ^a,b^	54.4 ± 7.3 ^a,c^	74.4 ± 32.8 ^b,c^	88.9 ± 41.1 ^b^	128.9 ± 10.5 ^d^
	Overall	108.9 ± 26.5 ^a^	47.8 ± 13.9 ^b^	61.7 ± 27.5 ^b^	102.8 ± 37.7 ^a^	115.0 ± 20.6 ^a^

^a–c^ Within a row, RMS values with a common superscript letter indicate no statistically significant difference (*p* > 0.05). RMS, root mean square; OM, outer measurement; IM, inner measurement.

**Table 3:** Statistical comparison of overall pre- and post-sterilization precision.

PRE	PolyJet Matte	PolyJet Glossy	SLA-LT	SLA-Luxa	SLA-NextDent
PolyJet Matte					
PolyJet Glossy	0.08				
SLA-LT	0.14	0.99			
SLA-Luxa	0.99	0.06	0.09		
SLA-NextDent	0.82	<0.05 *	<0.05 *	0.91	
**POST**	**PolyJet Matte**	**PolyJet Glossy**	**SLA-LT**	**SLA-Luxa**	**SLA-NextDent**
PolyJet Matte					
PolyJet Glossy	0.32				
SLA-LT	0.06	<0.05 *			
SLA-Luxa	0.43	<0.05 *	0.85		
SLA-NextDent	0.99	0.15	0.15	0.67	

* *p < 0.05* suggests a statistically significant difference between the indicated groups. Pre, pre-sterilization; Post, post-sterilization.

**Table 4:** Post-sterilization RMS values (mean ± SD) (µm) of precision and trueness for outer, inner, and overall measurements of test bodies fabricated using different biocompatible resin materials.

		PolyJet Matte	PolyJet Glossy	SLA-LT	SLA-Luxa	SLA-NextDent
	Measurement	Mean ± SD	Mean ± SD	Mean ± SD	Mean ± SD	Mean ± SD
Precision	OM	50.8 ± 29.7 ^a^	38.0 ± 24.7 ^a,b^	16.8 ± 12.0 ^b^	35.3 ± 15.8 ^a,b^	46.0 ± 19.4 ^a^
	IM	17.5 ± 11.1 ^a^	70.5 ± 8.0	20.5 ± 17.6 ^a^	10.3 ± 6.4 ^a^	17.8 ± 14.5 ^a^
	Overall	37.5 ± 28.9 ^a,b^	51.0 ± 25.4 ^b^	18.3 ± 14.2 ^a^	25.3 ± 17.8 ^a^	34.7 ± 22.3 ^a,b^
Trueness	OM	186.7 ± 18.0 ^a^	138.9 ± 11.7 ^b^	72.2 ± 19.9	127.8 ± 33.1 ^b^	158.9 ± 27.6 ^a,b^
	IM	81.1 ± 19.0 ^a,c^	214.4 ± 15.1	90.0 ± 25.5 ^b,c^	61.1 ± 22.1 ^a^	76.7 ± 20.0 ^a,b^
	Overall	133.9 ± 57.2 ^a^	176.7 ± 41.0	81.1 ± 23.9 ^b^	94.4 ± 43.8 ^a,b^	117.8 ± 48.3 ^a,b^

^a–c^ Within a row, RMS values with a common superscript letter indicate no statistically significant difference (*p* > 0.05). RMS, root mean square; OM, outer measurement; IM, inner measurement.

**Table 5:** Statistical comparison of overall pre- and post-sterilization trueness.

PRE	PolyJet Matte	PolyJet Glossy	SLA-LT	SLA-Luxa	SLA-NextDent
PolyJet Matte					
PolyJet Glossy	<0.05 *				
SLA-LT	<0.05 *	0.52			
SLA-Luxa	0.95	<0.05 *	<0.05 *		
SLA-NextDent	0.96	<0.05 *	<0.05 *	0.64	
**POST**	**PolyJet Matte**	**PolyJet Glossy**	**SLA-LT**	**SLA-Luxa**	**SLA-NextDent**
PolyJet Matte					
PolyJet Glossy	<0.05 *				
SLA-LT	<0.05 *	<0.05 *			
SLA-Luxa	0.61	<0.05 *	0.89		
SLA-NextDent	0.8	<0.05 *	0.15	0.52	

* *p* < 0.05 suggests a statistically significant difference between the indicated groups. Pre, pre-sterilization; Post, post-sterilization.

## Supplementary Materials

The following are available online at https://www.mdpi.com/2077-0383/9/5/1506/s1, Table S1: Pre- and post-sterilization percentage change in dimensions of test bodies.

## Abbreviations

3D	Three-dimensional
CAD	Computer-aided design
AM	Additive manufacturing
SLA	Stereolithography
UV	Ultraviolet
LT	Dental LT Clear
Luxa	LuxaPrint Ortho Plus
NextDent	NextDent Ortho Clear
IPA	Isopropyl alcohol
RMS	Root mean square
SD	Standard deviation
STL	Standard tessellation language
OM	Outer measurement
IM	Inner measurement
PLA	Polylactic acid
PDMS	Polydimethylsiloxane
PTEG	Polyethylene Terephthalate Glycol
UV	Ultraviolet
