# Supplementary material for: Effects of Steam Sterilization on 3D Printed Biocompatible Resin Materials for Surgical Guides—An Accuracy Assessment Study
Source: J Clin Med. 2020 May 17;9(5):1506. doi: 10.3390/jcm9051506 (PMC7291001; doi:10.3390/jcm9051506)
Supplement: Supplementary file 1 [file jcm-09-01506-s001.pdf]

Journal of
Clinical Medicine

Supplementary Materials: Effects of steam sterilization on 3D printed biocompatible resin materials for surgical guides – an accuracy assessment study

Neha Sharma [1,2], Shuaishuai Cao [1,2], Bilal Msallem [1,2], Christoph Kunz [1], Philipp Brantner [2,3], Philipp Honigmann [2,4,†], Florian M. Thieringer [1,2,*,†]

1 Department of Oral and Cranio-Maxillofacial Surgery, University Hospital Basel, Spitalstrasse 21, Basel, 4031, Switzerland; neha.sharma@usb.ch (N.S.); shuaishuai.cao@unibas.ch (S.C.); bilal.msallem@usb.ch (B.M.); christoph.kunz@usb.ch (C.K.); florian.thieringer@usb.ch (F.M.T.)
2 Medical Additive Manufacturing Research Group, Department of Biomedical Engineering, University of Basel, Gewerbestrasse 16, Allschwil, 4123, Switzerland; philipp.brantner@usb.ch (P.B.)
3 Radiology Department, University Hospital Basel, Petersgraben 4, Basel, 4031, Switzerland
4 Hand Surgery, Cantonal Hospital Basel-land, Rheinstrasse 26, Liestal, 4410, Switzerland; philipp.honigmann@ksbl.ch (P.H.)
* Correspondence: florian.thieringer@usb.ch
† These authors contributed equally to this work.

Received: 17 April 2020; Accepted: 14 May 2020; Published: 17 May 2020

S.1. Pre- and post-sterilization percentage change in dimensions of test bodies

The pre- and post-sterilization percentage (%) change in dimensions of test bodies in OM and IM measurements are displayed in Table S1. The results showed that although the post-sterilization percentage change in dimensions were higher than pre-sterilization counterparts (except SLA-Luxa), the relative change in dimensions with sterilization in reference to CAD dimensions from pre- to post-sterilization measurements were comparatively less in all groups.

Table S1. Pre- and post-sterilization percentage change in dimensions of test bodies.

Test body Measurement		PolyJet Matte		PolyJet Glossy		SLA-LT		SLA-Luxa		SLA-NextDent	
		Pre	Post	Pre	Post	Pre	Post	Pre	Post	Pre	Post
T1	OM	0.36	0.48	0.12	0.66	0.14	0.18	0.10	0.35	0.62	0.33
	IM	0.41	0.16	0.35	1.39	0.12	0.17	0.27	0.05	0.12	0.62
T2	OM	0.40	0.51	0.17	0.53	0.10	0.22	0.02	0.26	0.44	0.22
	IM	0.22	0.28	0.38	1.28	0.00	0.33	0.55	0.18	0.23	0.72
T3	OM	0.48	0.56	0.10	0.51	0.00	0.26	0.06	0.32	0.63	0.28
	IM	0.05	0.48	0.34	1.31	0.37	0.06	0.26	0.16	0.23	0.77
T4	OM	0.47	0.56	0.09	0.54	0.14	0.34	0.10	0.23	0.71	0.47
	IM	0.14	0.52	0.38	1.37	0.39	0.03	0.45	0.20	0.34	0.86
T5	OM	0.52	0.65	0.04	0.57	0.07	0.16	0.11	0.11	0.68	0.26
	IM	0.02	0.39	0.29	1.46	0.54	0.52	0.81	0.44	0.51	0.82
T6	OM	0.50	0.63	0.01	0.40	0.01	0.21	0.08	0.23	0.65	0.29
	IM	0.29	0.05	0.25	1.20	0.38	0.09	0.56	0.27	0.29	0.75
T7	OM	0.47	0.53	0.05	0.51	0.04	0.34	0.01	0.43	0.85	0.50
	IM	0.16	0.22	0.26	1.18	0.41	0.36	0.46	0.06	0.30	0.58
T8	OM	0.45	0.62	0.01	0.59	0.02	0.19	0.09	0.22	0.64	0.23
	IM	0.20	0.16	0.30	1.29	0.31	0.09	0.45	0.33	0.30	0.62
T9	OM	0.53	0.71	0.02	0.51	0.02	0.32	0.02	0.27	0.99	0.53
	IM	0.10	0.54	0.33	1.44	0.33	0.36	0.14	0.22	0.27	0.77

Overall % change in dimensions	0.32	0.45	0.19	0.93	0.19	0.23	0.25	0.24	0.49	0.53
